# Contrasting Pollinators and Pollination in Native and Non-Native Regions of Highbush Blueberry Production

**DOI:** 10.1371/journal.pone.0158937

**Published:** 2016-07-08

**Authors:** Jason Gibbs, Elizabeth Elle, Kyle Bobiwash, Tiia Haapalainen, Rufus Isaacs

**Affiliations:** 1 Department of Entomology, Michigan State University, East Lansing, Michigan, United States of America; 2 Department of Biological Sciences, Simon Fraser University, Burnaby, British Columbia, Canada; University of Guelph, CANADA

## Abstract

Highbush blueberry yields are dependent on pollination by bees, and introduction of managed honey bees is the primary strategy used for pollination of this crop. Complementary pollination services are also provided by wild bees, yet highbush blueberry is increasingly grown in regions outside its native range where wild bee communities may be less adapted to the crop and growers may still be testing appropriate honey bee stocking densities. To contrast crop pollination in native and non-native production regions, we sampled commercial ‘Bluecrop’ blueberry fields in British Columbia and Michigan with grower-selected honey bee stocking rates (0–39.5 hives per ha) to compare bee visitors to blueberry flowers, pollination and yield deficits, and how those vary with local- and landscape-scale factors. Observed and Chao-1 estimated species richness, as well as Shannon diversity of wild bees visiting blueberries were significantly higher in Michigan where the crop is within its native range. The regional bee communities were also significantly different, with Michigan farms having greater dissimilarity than British Columbia. Blueberry fields in British Columbia had fewer visits by honey bees than those in Michigan, irrespective of stocking rate, and they also had lower berry weights and a significant pollination deficit. In British Columbia, pollination service increased with abundance of wild bumble bees, whereas in Michigan the abundance of honey bees was the primary predictor of pollination. The proportion of semi-natural habitat at local and landscape scales was positively correlated with wild bee abundance in both regions. Wild bee abundance declined significantly with distance from natural borders in Michigan, but not in British Columbia where large-bodied bumble bees dominated the wild bee community. Our results highlight the varying dependence of crop production on different types of bees and reveal that strategies for pollination improvement in the same crop can vary greatly across production regions.

## Introduction

There is currently great interest in developing farm management practices to support sustainable crop pollination strategies that maximize yields. This is driven in part by concerns over the long-term availability and cost of managed bees, particularly the honey bee (*Apis mellifera* L.), and by evidence for declines in the populations of some wild bee species from regions of agricultural production [1]. At intensively managed farms, honey bee hives are brought by beekeepers to flood fields with high densities of pollinators for the short period of bloom. As a result, honey bees are typically the most abundant pollinator on farms and provide valuable pollination services to fruit, vegetable, and nut crops [2]. Wild bees also contribute to crop pollination, but where agriculture dominates land use, there may be a depauperate community of wild bees due to the loss of habitat used for nesting and for foraging before or after crops bloom [3,4]. In contrast, diversified crop systems and those set within diverse landscapes have more abundant wild bee communities that can contribute to higher levels of crop pollination [5].

Highbush blueberry (*Vaccinium corymbosum* L.) requires insect-mediated pollination for productive yields [6,7], with deposition of compatible pollen onto stigmas during bloom leading to increased fruit set and berry size [8]. The structure of blueberry anthers is such that pollen is most effectively released by active vibration of flowers [9]. Buzz-pollination, the behavior of vibrating flowers to release pollen, is performed by a host of wild bee species, but not by honey bees [10,11]. Honey bees can still pollinate blueberry, but tend to have a lower per-visit pollen deposition rate than some other species [12]. To achieve pollination, growers raise or rent honey bee colonies and stock their fields with a range of colony densities [13,14], ranging from no use of honey bees to 39.5 colonies per ha (this study). The majority of growers use 6.2–12.3 colonies per ha, which is expected to be sufficient for adequate pollination [6,15]. This wide range of investment in managed bees can reflect grower attitudes regarding the ability of wild bees to pollinate the crop and their interest in ensuring that pollination is not limiting to their blueberry yields. However, the degree of pollen limitation is not well understood in commercial blueberry production, so there may be missed opportunities for greater yield that additional bee abundance and/or diversity could support. Recent independent studies in British Columbia and Michigan, two major regions of highbush blueberry production, provide insights into the potential for improving pollination. Button and Elle [16] detected pollen limitation in British Columbia blueberry fields, but also found that bumble bees helped reduce the levels of that deficit. In Michigan, Isaacs and Kirk [15] did not measure limitation *per se*, but large commercial fields with high levels of honey bee stocking had greater fruit set and berry size than small, less intensively managed fields. The bee community in those smaller Michigan fields had most of the pollination services delivered by wild bees. In this study, we applied consistent experimental protocols and sampling methods to investigate blueberry pollination in both regions. We determined the relative role of honey bees and wild bees in large-scale commercial blueberry fields and determined whether pollination was limited by insufficient wild bees, honey bees or both. The cross-region comparison also provided a range of landscape conditions for testing whether local or landscape scale features around fields explain the level of contribution to pollination by wild bees. Bees vary in their foraging range [17,18] and distribution [19] so examining blueberry-visiting bees over multiple regions and landscape scales can help reveal how these factors affect pollinators and their pollination services.

Understanding the relative importance of honey bees and wild bees to agricultural production provides the basis for pollination management decisions, and our research aims to improve blueberry pollination through investigation of the interactions between farm landscapes, bees, and crops. Here, we report on a study in that aimed to 1) determine how bee visitation and supplemental pollination affect blueberry fruit set, seed set, berry weight and yield; 2) determine how the bee community at each field affects the level of blueberry pollination; and 3) examine how local- and landscape-scale factors affect bee abundance-diversity patterns and the level of blueberry pollination and yield. The research was conducted in two primary North American regions of highbush blueberry production, Michigan and British Columbia, providing insights across a range of fields with different landscape contexts and levels of stocking with honey bees.

## Methods

### Site selection

Seventeen privately owned commercial fields of *V*. *corymbosum*, cultivar “Bluecrop”, were selected in both British Columbia and Michigan, for a total of 34 fields (S1 Table). Landowner permission was acquired to conduct research on each field. All fields were selected to be adjacent to semi-natural habitat, typically woods or scrub-land. Semi-natural borders were of various sizes, in a few cases comprising only a narrow strip of trees and unmanaged vegetation. Fields were selected to be more than 5 ha, with sufficient area of Bluecrop cultivar to sample a depth extending 100 m into the field, and with additional blueberries extending beyond that distance. Four transects were laid out in each field running parallel to the border, at distances of 0, 25, 50, and 100 m from the field edge. At two fields in Michigan, a change in cultivar occurred between the 50 and 100 m distances. The 100 m distance for one field was ultimately excluded on this basis and in the other field a replacement transect at approximately 75 m was used instead.

### Flower-visitor abundance and diversity

Observations of all flower-visiting insects (hereafter “flower-visitors”) were made during 10 min timed samples in each of the four transects. Each field was visited 2 (in British Columbia) or 3 (in Michigan) times during weather conditions suitable for bee flight (>15.5°C, low wind speeds, at least partial sun, no precipitation). Fields were visited at different times of the day and in different orders to limit temporal biases. We categorized flower-visitors into four categories: honey bees (*Apis mellifera*), bumble bees (genus *Bombus*), other bees (primarily *Andrena*, *Ceratina*, *Osmia* or halictid bees) and other flower-visitors (predominantly flies in the family Syrphidae). Bees and syrphid flies are typically considered the most important pollinating taxa [20].

During the same sample date as the observations all insect flower-visitors, excluding *A*. *mellifera*, were collected using aerial nets for 10 min per transect. All collected specimens were killed, pinned, labeled, databased, and identified to species or morpho-group by comparison to voucher material in collections (Michigan State University—A.J. Cook Arthropod Research Collection, Simon Fraser University—Elizabeth Elle Research Collection, and Jason Gibbs personal collection) and published revisions and keys [21–37].

We compared bee richness and total visitor richness between the two regions. All analyses were performed in R [38]. Due to very low abundances of non-bee flower-visitors, we focused our analyses on the bee communities. We examined differences between regions using species accumulation curves and Chao-1 richness estimator [39–41] implemented using the *vegan* package [42]. *Apis mellifera* were not included in the species accumulation curves. The flower-visiting communities of our two regions were expected to be quite distinct, although a small proportion of the species collected are known to have transcontinental distributions. We also calculated Shannon-Wiener diversity indices for our sites [43] and compared regions using a Mann Whitney (M-W) test. We also used non-metric multidimensional scaling implemented in the *vegan* package to compare Bray Curtis dissimilarity of farms and regions and landscape factors affecting community composition. We used PERMANOVA to compare community composition between regions.

### Factors affecting flower-visitor abundance and diversity

**Fig 1:**
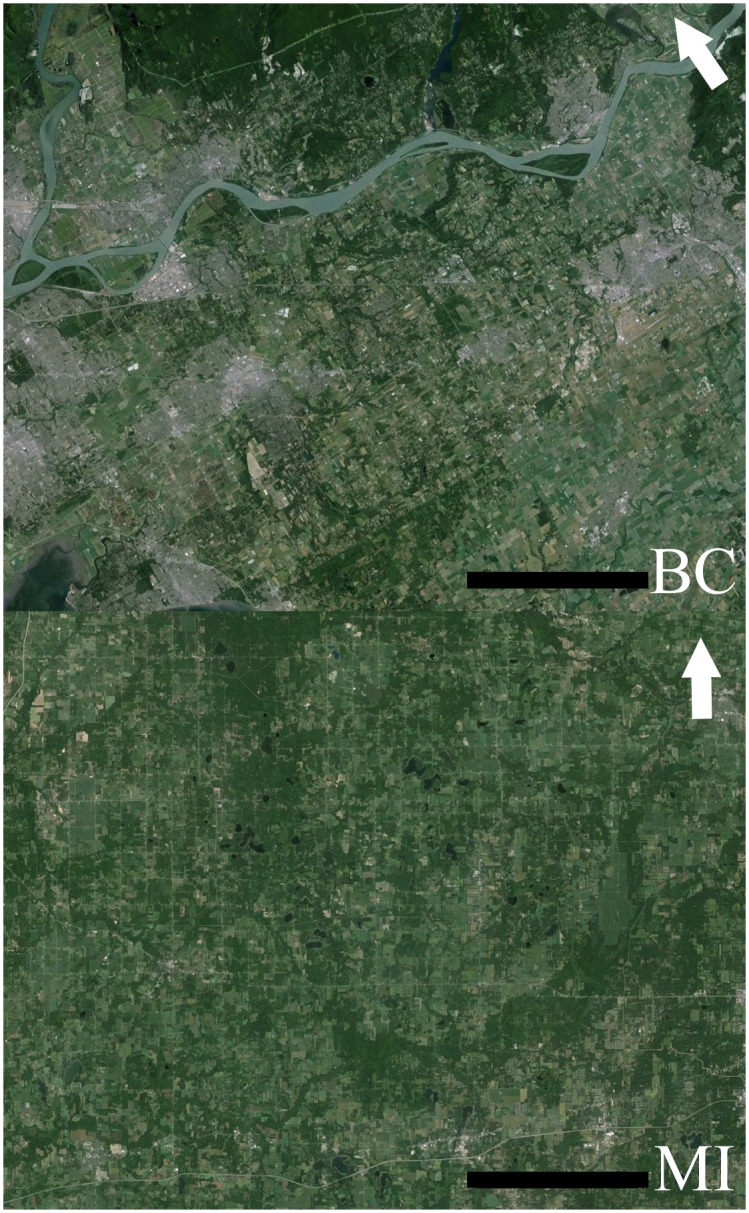
Landscape context of British Columbia (BC) and Michigan (MI) blueberry fields. Google Earth^™^ images showing landscapes surrounding blueberry fields (not shown) in British Columbia and Michigan. The maps encompass all 17 blueberry fields in BC and 12 of 17 fields in MI. The area encompassing the remaining five MI fields is not shown to maintain a consistent scale (scale bars equal 10 km). The maps show greater proportion of urban development and agriculture in BC. Arrows indicate the direction of north.

**Fig 2:**
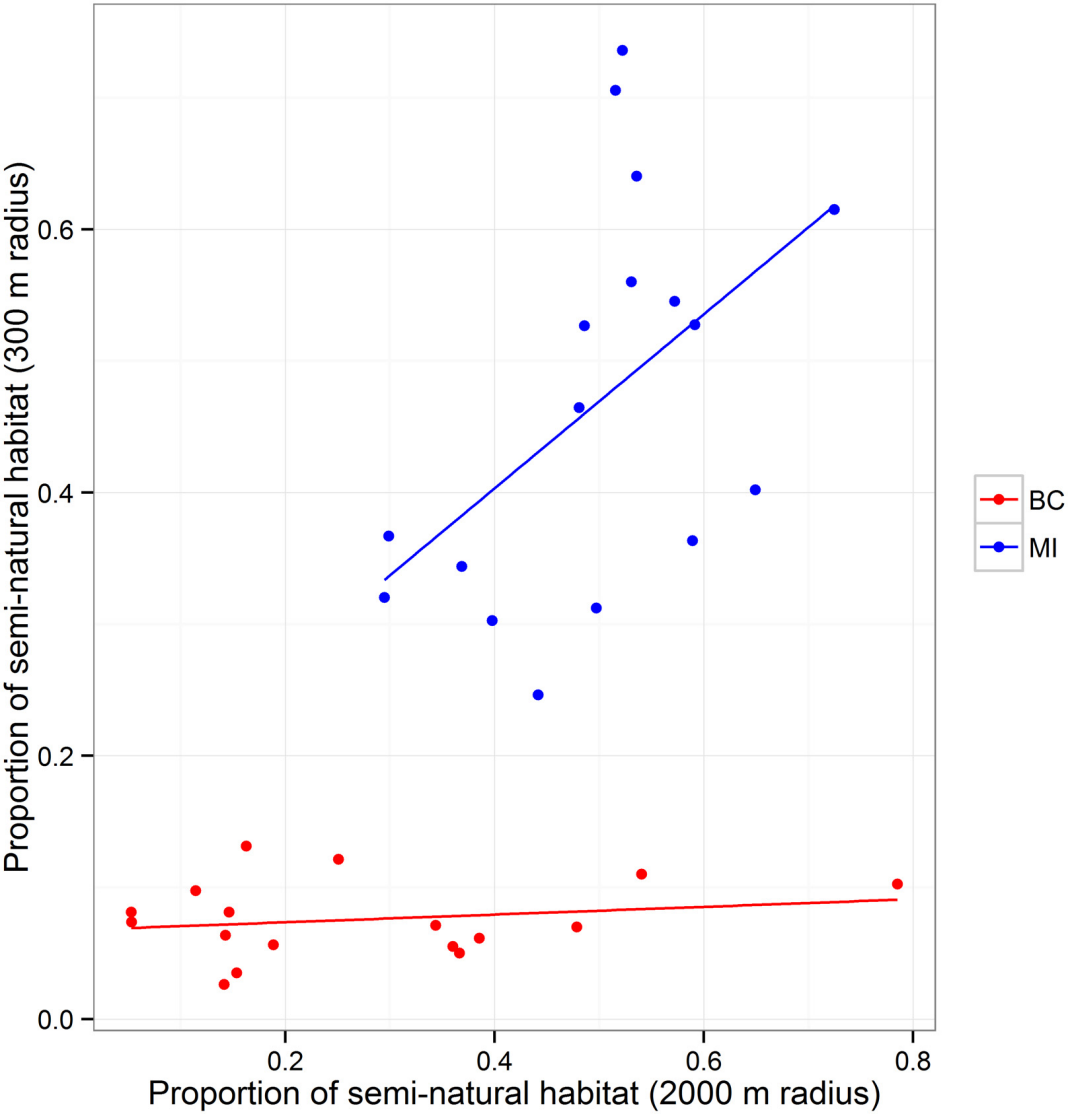
Extent and relationship of semi-natural habitat at 300 m and 2000 m radii surrounding British Columbia (BC) and Michigan (MI) blueberry fields. The extent of semi-natural habitat in the landscape surrounding British Columbia blueberry fields is often much less than blueberry fields in Michigan. The proportions of semi-natural habitat at different landscape scales (300 m and 2000 m radii) are not correlated in British Columbia, but show a strong positive correlation in Michigan.

To determine the effect of landscape composition on the bee communities recorded in each of the fields, GPS coordinates were taken at the center of the 0 m transect for each field and the land use within 300 m of this point was hand-digitized using aerial maps in ArcGIS (ESRI Inc., Redlands, CA) at a scale of 1:2000. The maps were categorized into 26 standardized land cover classes (S2 Table). We used a 300 m scale because this is a typical foraging range for many of the smaller wild bee species [17,18]. We also created circles with 1000 and 2000 m radii around each field using land cover layers available for the United States (USDA National Agricultural Statistics Service Cropland Data Layer 2013) and Canada (GeoBase Canada, Natural Resources Canada), to quantify the larger landscape scale that colony-forming bees are capable of foraging in [17,18]. Due to differences in land use categorization between the two data sets, we grouped land cover classes into two categories, either “semi-natural” or “disturbed”, based on level of anthropogenic disturbance and expected availability of pollinator habitat. Urban areas, mowed areas and agricultural lands were the primary “disturbed” habitats. Woodlands, unmowed grasslands and marshes were included in the “semi-natural” areas. We then used the land cover data to determine the proportion of semi-natural habitat in the surrounding landscape (Figs 1 and 2).

To examine factors related to bee abundance we created generalized linear mixed models (GLMMs), with one of the four flower-visitor groups (*Apis*, *Bombus*, other bees, other flower-visitors) as the response variable, using proportion of semi-natural habitat, distance into field (0, 25, 50 and 100 m), date, time of day and the remaining flower-visiting groups as potential explanatory variables. Distance was treated as a fixed factor and sample data and time were treated as random effects. We examined the proportion of semi-natural habitat at three different scales (300, 1000 and 2000 m radii) and tested for autocorrelation between our explanatory variables before selecting which ones to include in each analysis. We also included an interaction term for distance and proportion of semi-natural habitat. When modeling abundances of specific visitor categories (e.g. bumble bees), other flower-visiting groups were included in the model only if they were not correlated with other explanatory variables. Honey bee colony stocking rate was obtained from growers and included as an explanatory variable for honey bee abundance regardless of results from autocorrelation tests.

Linear models with a negative binomial error distribution were made using the *glmmADMB* package [44]. The Akaike Information Criterion, with small sample correction (AIC_c_), was used in model selection, with the *MuMIn* package [45]. Models with delta AIC_c_ values within 2 units of the best model are also considered to have substantial support [46]. When examining the response of bee communities to land use factors, we chose the radius which produced the lowest AIC_c_ values in conjunction with other variables (e.g. honey bee stocking rates). When multiple models showed substantial support, model-averaged parameter estimates were made using the *MuMIn* package.

### Blueberry pollination and pollen limitation

During spring 2013, at each of the four transects in each field, we selected 10 blueberry bushes (670–680 bushes total per region; one transect from Michigan was excluded because it was a different cultivar) and haphazardly selected 3 flower clusters of similar developmental stage on second year canes from each bush (2010–2040 clusters total per region). For each of these clusters, the number of flowers was counted, and the clusters on each bush were assigned to one of three treatments: pollinator exclusion (hereafter “exclusion”), fully accessible to insects (hereafter “open”) or fully accessible with supplemental pollen applied to the stigma by hand (hereafter “hand-supplemented pollination”). In the exclusion treatment a group of clusters from each bush was covered with a 1 gallon mesh bag (The Cary Company, Addison, IL) prior to flower opening to exclude insect flower-visitors, and sealed onto the branch using a twist tie [47,48]. For the hand-supplemented pollination treatment, when individual flowers were open, additional pollen was collected from a nearby bush of the same cultivar and applied to the stigma. An electric toothbrush was placed on the corolla to vibrate pollen out of donor flowers into a Petri dish, and then a small paint brush was used to immediately apply pollen directly to the stigma of the hand-supplemented pollination flowers [16]. Due to the sequential opening of blueberry flowers, this was performed every 4–5 days, weather permitting, resulting in supplemental pollen being added 3 (in Michigan) and 5 (in British Columbia) times on each hand-supplemented pollination cluster. After bloom was complete, the hand-supplemented pollination and open clusters were also bagged to prevent herbivory, pest infestation, premature harvesting, and to standardize conditions among treatments during fruit development.

The clusters were harvested when more than 50% of the berries had become ripe. This gives a result consistent with harvesting individual berries from a cluster over time [49], while minimizing the risk of berries dropping off or becoming moldy. Collected berries were frozen until measurements could be taken and then the berries from each cluster were counted and weighed to determine effects of pollination treatments on fruit set and average berry weight. We then subsampled four bushes from each transect at random (bushes in which one pollinator treatment was lost, damaged or prematurely harvested were excluded prior to the random selection). Three ripe berries from these clusters were individually weighed and the number of mature seeds counted following the criteria of Desjardins and De Oliveira [50]. We then examined the effects of pollination treatment and flower-visitor diversity on the weight and seed set of these focal berries.

The average fruit set, seed set and berry weights measured in each field were used to test for differences among pollination treatments and the effect of distance on the open pollination treatment. Percent fruit set was calculated for each cluster by dividing the number of developing fruit by the number of flowers counted earlier in the season. The non-parametric Kruskal-Wallis (K-W) test was used to determine the significance of differences among treatments due to non-normality in some of the data, specifically the pollinator excluded treatment, as determined using Shapiro-Wilk tests. We then used the Mann-Whitneytest for all pair-wise comparisons followed by a Tukey honest significant difference (HSD) test to contrast means.

Generalized linear models were used to analyze effects of honey bees, wild bees and pollinator diversity on the response variables of fruit set, seed set and berry weight due to pollination. Pollinator contributions to fruit set, seed set and berry weight were calculated by subtracting the values measured in the pollinator exclusion treatment from the values obtained from the open pollination treatment. Shapiro-Wilk tests were used to test normality of the response variables and normality of model residuals.

We calculated pollination deficits by subtracting measurements in the open pollination treatment from those of the hand-supplemented pollination treatment, which should maximize fruit set, seed set and berry weight. These calculations were done for each bush and then the average differences were determined for each field. The proportion of potential yield lost per field was then calculated from the weight deficit values.

### Economic benefits of pollination

The economic values of pollinator contributions to yield in both regions were determined by estimating the yield per hectare in each field and multiplying that by the mean farm gate price for blueberry in both regions during 2013 [16]. To estimate the total number of berries per hectare we first estimated the number of bushes per hectare from the bush and row spacing in each field. The number of canes per bush was averaged from counts on each of our 40 focal bushes per field. To determine the number of flowers per bush, a single cane from each bush was haphazardly selected and the number of clusters was counted. The number of fruit on two clusters was also counted and then averaged for each of the 40 selected canes. The total berries per hectare was then calculated using the following equation:

$$\frac{Berries}{ha}=\frac{Bushes}{ha}x\frac{Canes}{Bush}x\frac{Clusters}{Cane}x\frac{Berries}{Cluster}$$ (1)

Yield was estimated in kg/ha for the three pollination conditions (*Y*_*o*_−open pollination, *Y*_*s*_−hand-supplemented pollination and *Y*_*e*_−pollinator exclusion) using berries/ha values from Eq 1 and the mean fruit weight (*W*) and fruit set (*FS*) from each field for each pollination condition.

Yield, measured in kilograms of blueberries per hectare, was calculated using Eq 2:

$$Yield\;=\left(\frac{Berries}{ha}x\;W_{o}\right)$$ (2)

where *W*_*o*_ is equal to the average berry weight from the open pollination treatment.

To calculate the amount of yield that is due to pollination we incorporated fruit set and berry weight from both the open and pollinator excluded treatments in Eq 3:

$$Y_{p}=\left(\frac{Berries}{ha}x\;Wo\right)-\left(\frac{Berries}{ha}x\;\left(\frac{\;FS_{e}}{FS_{o}}\right)x\;W_{e}\right)$$ (3)

where *Y*_*p*_ is the amount of yield (kg/ha) due to pollination, *FS*_*e*_ is the fruit set in the excluded treatment, *FS*_*o*_ is the fruit set from the open pollination treatment, and *W*_*e*_ is the average berry weight from the pollinator excluded treatment.

To estimate the potential unrealized yield due to insufficient pollination, we used the fruit set and berry weight from the hand-supplemented and open treatments in Eq 4

$$Y_{d}=\;\left(\frac{Berries}{ha}x\;\left(\frac{\;FS_{s}}{FS_{o}}\right)x\;W_{s}\right)-\left(\frac{Berries}{ha}x\;W_{o}\right)$$ (4)

where *Y*_*d*_ is the amount of unrealized yield (kg/ha) that was not achieved due to pollination deficits and *FS*_*s*_ and *W*_*s*_ are the fruit set and average berry weight, respectively, from the hand-supplemented treatment.

In 2013, the mean farm gate price of blueberry sold directly from the farm was $3.30/kg (USD) in BC and $2.84/kg (USD) in Michigan. Yields from each pollination condition were multiplied by the appropriate price and then averaged across each pollination treatment. Values from each field were obtained and averaged for fields in both regions to determine the crop value attributable to measured pollination conditions and potential crop value attainable if pollinator abundance is enhanced.

## Results

### Flower-visitor abundance and diversity

**Fig 3:**
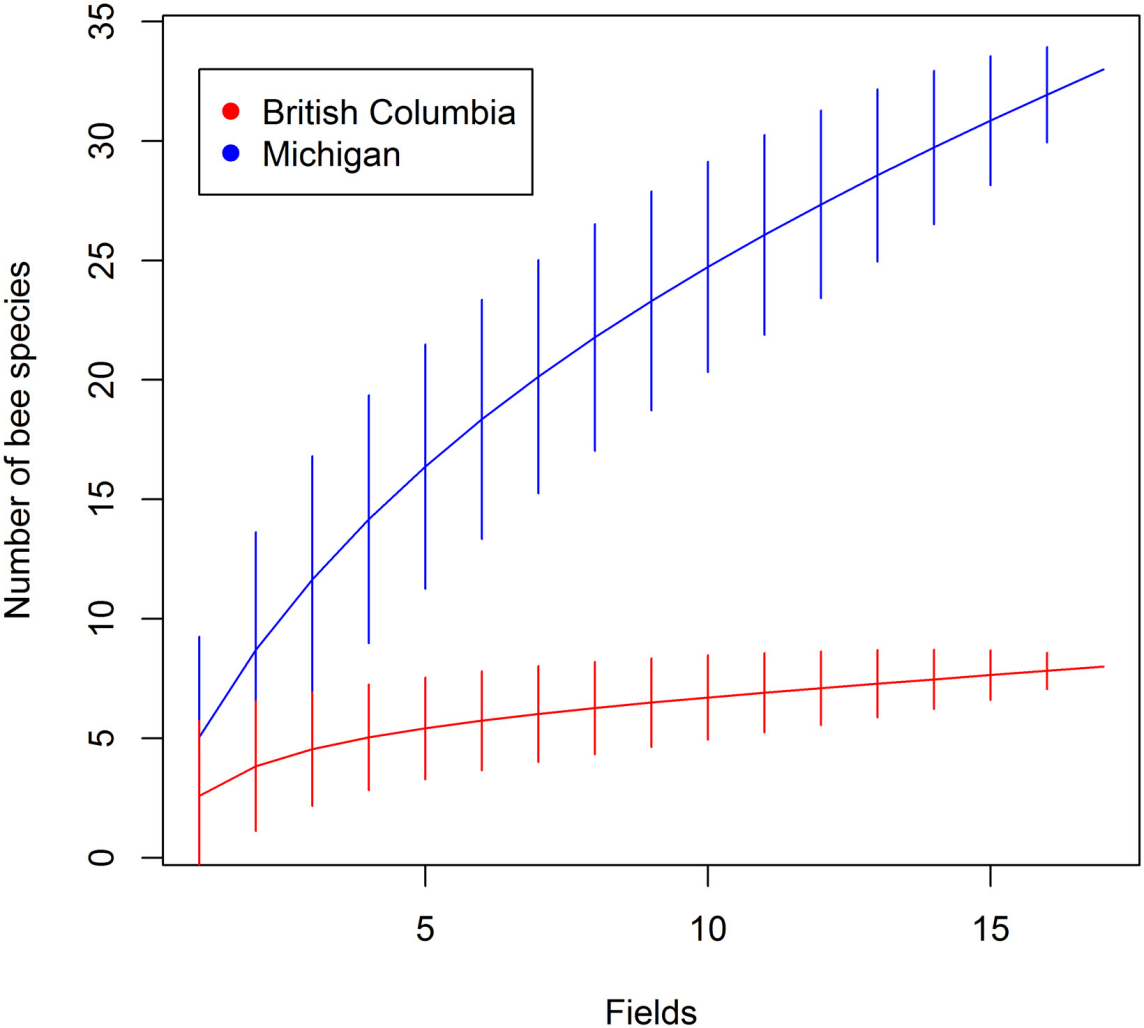
Species accumulation of blueberry flower visiting bees. Accumulated number of bee species from British Columbia (red) and Michigan (blue) blueberry fields estimated using rarefaction with confidence intervals of two standard deviations. Chao-1 richness estimates are 11 species for British Columbia and 71 species for Michigan (rounded to the nearest whole number).

Surveys of flower-visitors in highbush blueberry fields revealed greater bee species richness in Michigan fields than British Columbia, in terms of accumulated species richness for the region (Fig 3; S3 Table), average observed richness per field (two sample t-test, *t* = 3.77, df = 29.7, *p* < 0.001) and Chao-1 richness estimates (M-W, *U* = 37, *p* < 0.001).

**Fig 4:**
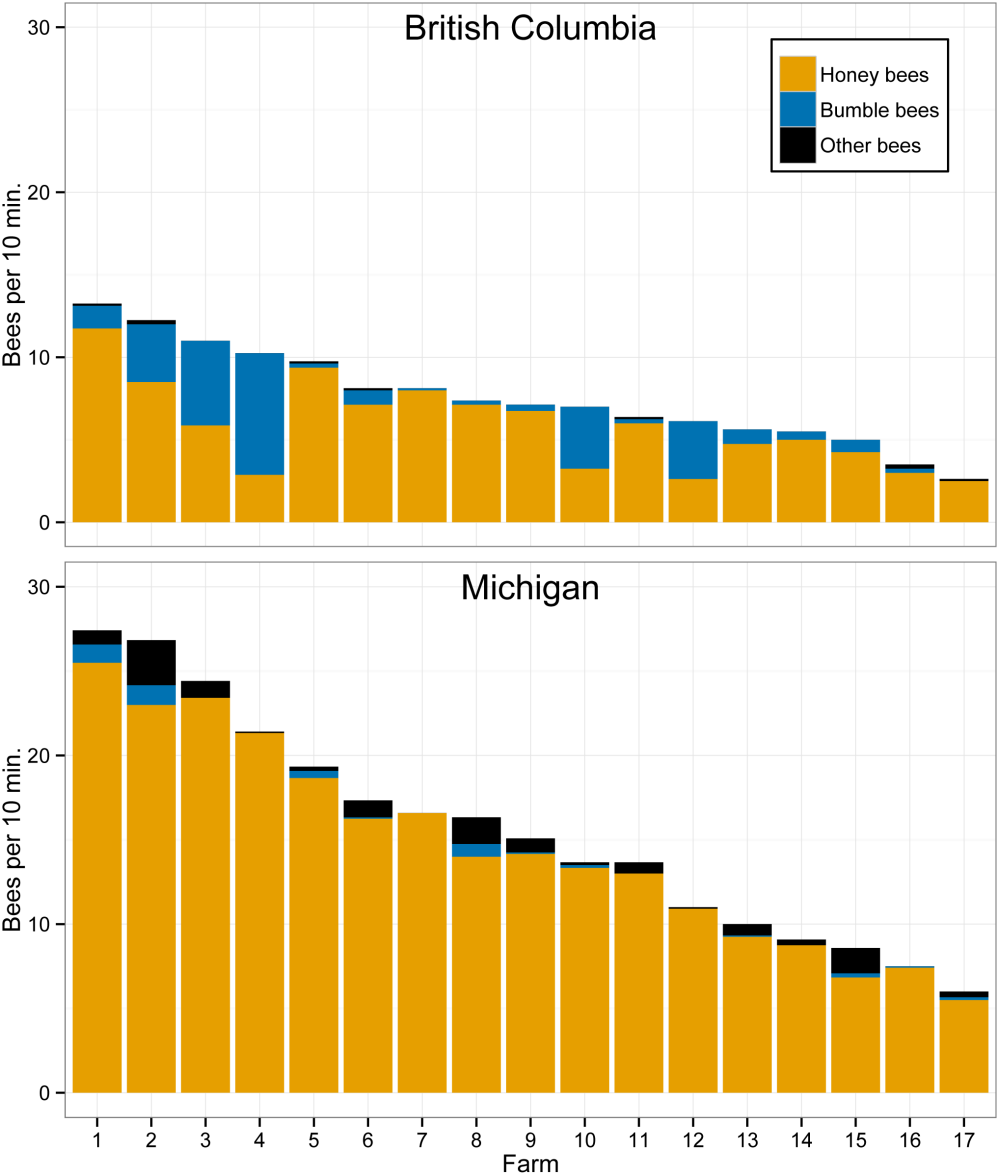
Relative bee abundance in highbush blueberry fields. Average bee abundance observed per ten-minute sampling period in highbush blueberry fields from British Columbia and Michigan grouped into three categories: honey bees, bumble bees and other bees.

**Fig 5:**
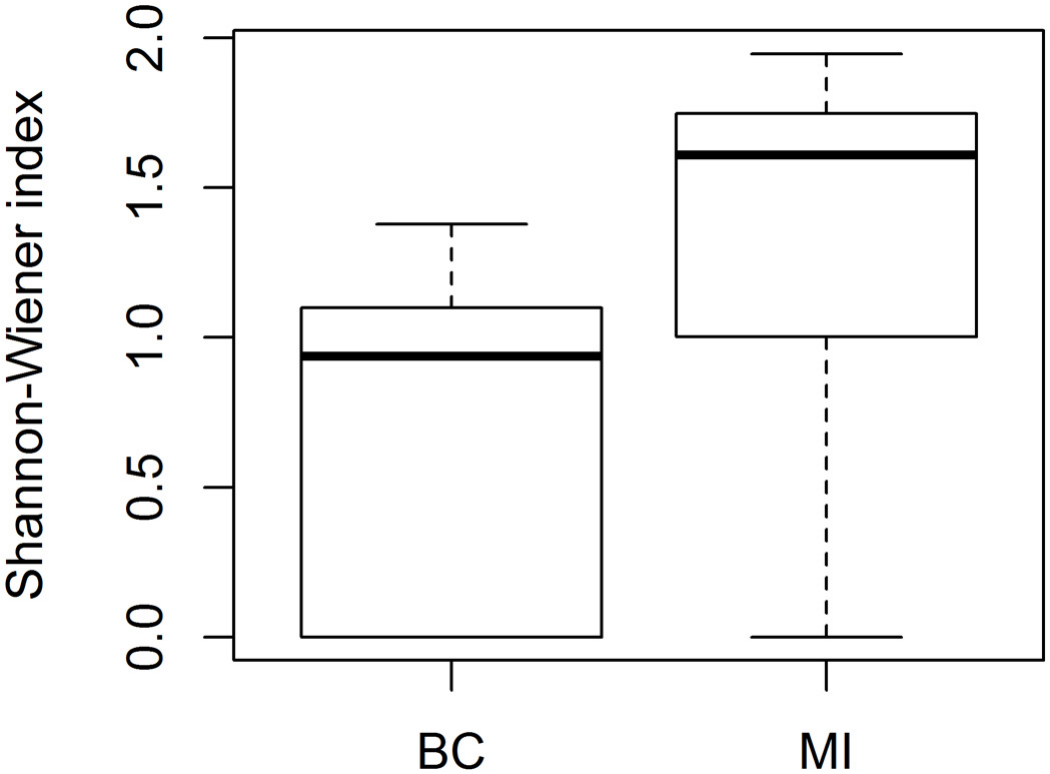
Shannon-Wiener diversity of blueberry visiting bees across regions. Field-based Shannon-Wiener diversity of bees for British Columbia (BC) and Michigan (MI). Boxplots are based on Shannon-Wiener diversity measures for each field. Boxes represent the inter-quartile range (25^th^ to 75^th^ percentile). The line represents the median and whiskers are the furthest data points within 1.5 times the inter-quartile range.

**Fig 6:**
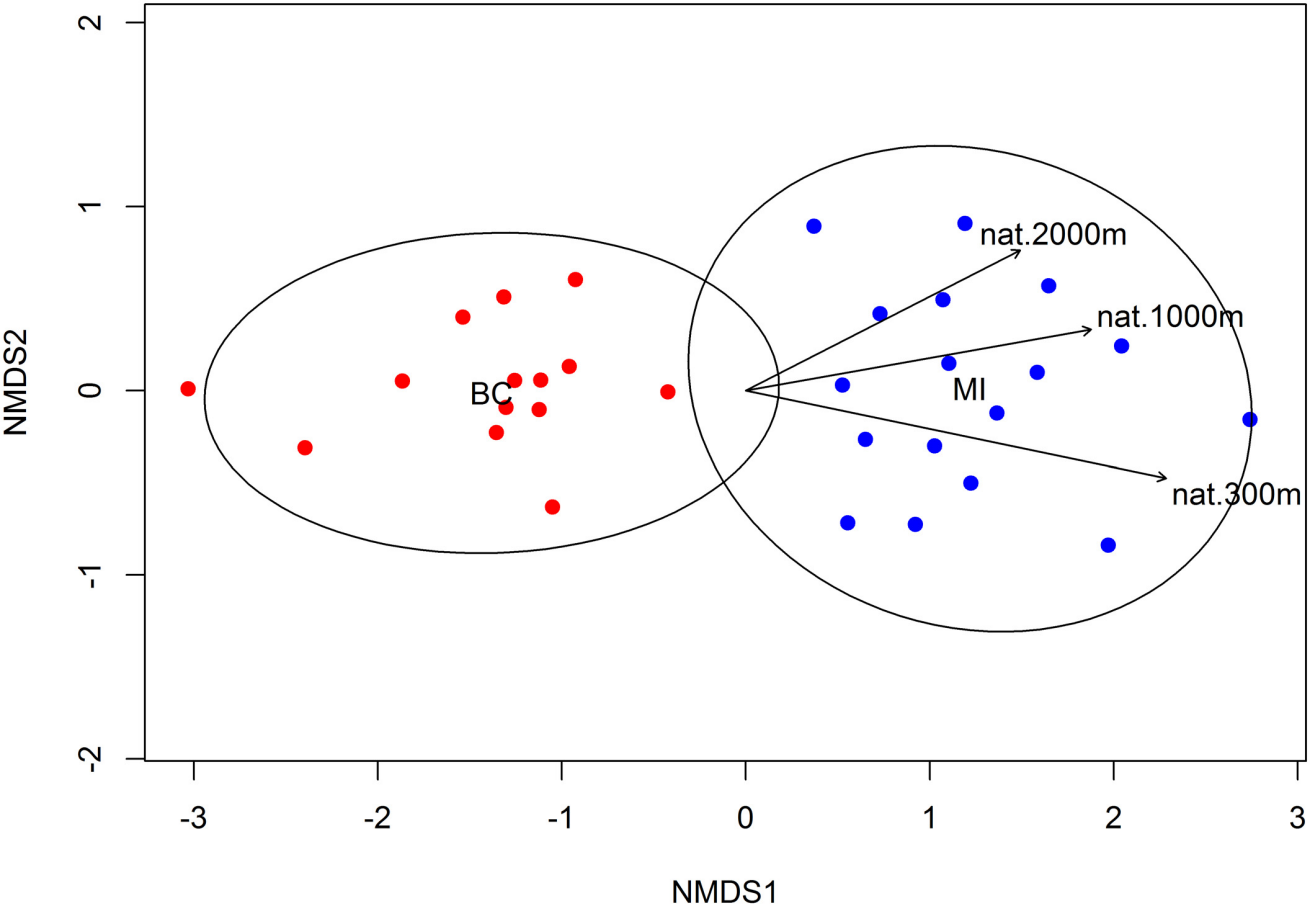
Non-metric multidimensional scaling using Bray Curtis dissimilarity distances. A distance matrix of bee community composition using the Bray Curtis index was used to form a non-metric multidimensional scaling plot. Fields that are closer in ordination space have more similar bee community composition. Ellipses represent groupings by region using 0.95 confidence interval. The arrows indicate the direction of increasing semi-natural habitat for 300 m, 1000 m and 2000 m radii in the ordination space for all fields. The length of arrows is proportional to the strength of the correlation of semi-natural habitat and the ordination.

Flower-visitor abundance varied across regions and fields, particularly the number of honey bees, with much greater honey bee abundance in Michigan blueberry fields (Fig 4). The bee fauna in Michigan was also more diverse when comparing Shannon-Weiner diversity (Fig 5, M-W, *U* = 59.5, *p* < 0.004). Community composition also differed significantly (Fig 6; PERMANOVA: *F* = 11.9, df = 1, *p* = 0.001) and there was also greater community compositional dissimilarity among Michigan fields (Fig 6). Community composition of the two regions were separated on the first NMDS axis, which also correlated strongly with land use (Fig 6).

### Factors affecting flower-visitor abundance and diversity

There was no significant difference in total flower-visitor or total bee abundance with distance from natural borders (total bees: K-W, χ^2^ = 1.3 (BC), 3.9 (MI), df = 3, *p* > 0.2, both regions). However, in Michigan, but not British Columbia, wild bees and the subset of wild bees excluding *Bombus* spp. varied significantly with distance from natural borders (K-W, χ^2^ = 27.3 (excluding *Bombus* χ^2^ = 33.8), df = 3, *p* < 0.0001 [both tests]), with wild bee abundance significantly reduced at 50 m (M-W, *U* = 239, Tukey HSD *p* < 0.007) and 100 m (M-W, *U* = 238, Tukey HSD *p* < 0.002) compared to bee abundance at field borders. Fruit set, seed set and berry weight did not vary significantly with distance from the field border in the open pollination treatment for either region (K-W; BC: fruit set: χ^2^ = 2.4, df = 3, *p* > 0.4; seed set: χ^2^ = 0.5, df = 3, *p* > 0.7; fruit weight: χ^2^ = 0.5, df = 3, *p* > 0.7; MI: fruit set: χ^2^ = 0.9, df = 3, *p* > 0.8; seed set: χ^2^ = 0.5, df = 3, *p* > 0.8; fruit weight: χ^2^ = 0.5, df = 3, *p* > 0.5).

**Fig 7:**
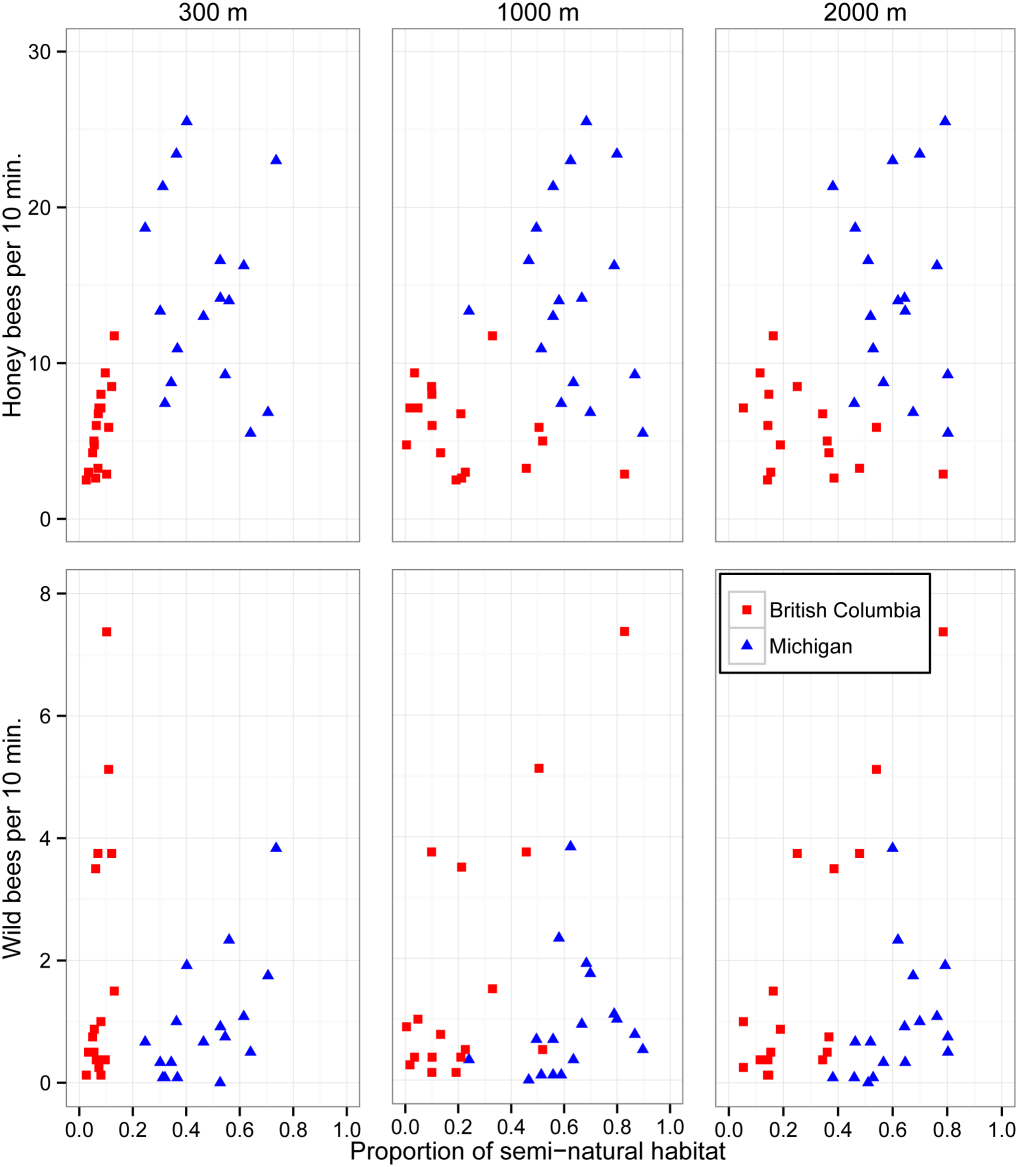
Land use effects on bee abundance. Relationship of proportion of semi-natural habitat surrounding blueberry fields in British Columbia (red squares) and Michigan (blue circles) to honey bee and wild bee abundance measured at three spatial scales (300 m, 1000 m, and 2000 m radii).

Land use surrounding blueberry fields in British Columbia and Michigan differed in the proportion of semi-natural habitat (Figs 1 and 2). In British Columbia, the abundance of wild bees, which is dominated by bumble bees, is positively related to the proportion of semi-natural habitat at the landscape scale (2000 m radius) (Tables 1 and 2; Fig 7). In Michigan, abundance of wild bees was positively related to the proportion of semi-natural habitat at the local scale (300 m radius) (Tables 1 and 2; Fig 7). Interestingly, we found for both regions that wild bee abundance inside fields was greater when rows were oriented perpendicular, rather than parallel, to the border (Tables 1 and 2). This suggests that wild bees are more likely to move into fields with the direction of rows.

**Table 1:** Model selection for factors affecting bee abundance in highbush blueberry fields of British Columbia (BC) and Michigan (MI). Only suboptimal models with ΔAIC_c_ ≤ 3 are shown.

Response	Region	Explanatory variables	df	logLik	AIC_c_	delta	Model weight
Wild bees	BC	Honey bees + Land use (2000 m) + Row orientation	9	-207.07	431.27	0	0.32
		Land use (2000 m) + Row orientation	7	-208.31	431.49	0.21	0.29
		Honey bees + Land use (2000 m)	7	-208.34	431.55	0.27	0.28
	MI	Distance from edge + Honey bees + Land use (300 m) + Row orientation	11	-226.74	476.88	0	0.52
		Distance from edge + Honey bees + Land use (300 m) + Row orientation + Orientation/distance interaction	14	-224.08	478.41	1.54	0.24
		Distance from edge + Land use (300 m) + Row orientation	10	-229.07	479.31	2.43	0.16
Honey bees	BC	Land use (300 m) + Row orientation	7	-345.20	705.27	0	0.40
		Land use (300 m)	6	-346.66	705.97	0.70	0.28
		Stocking rate + land use (300 m) + Row orientation	8	-345.18	707.49	2.22	0.13
		Stocking rate + land use (300 m)	7	-346.58	708.03	2.76	0.10
	MI	Land use (1000 m)	6	-717.93	1448.30	0	0.28
		Land use (1000 m) + Row orientation	7	-717.38	1449.34	1.04	0.17
		(null)	5	-719.74	1449.79	1.49	0.13
		Stocking rate + land use (1000 m)	7	-717.92	1450.42	2.12	0.11
		Row orientation	6	-719.16	1450.75	2.45	0.08

**Table 2:** Model averaged coefficients for significant (α ≤ 0.05) and marginally significant (α ≤ 0.1) factors affecting bee abundance on blueberry flowers during bloom in British Columbia (BC) and Michigan (MI), using sample day and time as random effects. The full average was used in all estimates and z value calculations.

Response	Region	Variable	Estimate	z value	p
Wild bees	BC	(Intercept)	-1.04 ± 0.55	1.88	0.060
		Land use (2000 m)	4.04 ± 0.60	6.62	**< 0.0001**
	MI	(Intercept)	-2.72± 0.51	5.28	**< 0.0001**
		Distance (25 m)	-0.88 ± 0.34	2.56	**0.01**
		Distance (50 m)	-1.54 ± 0.81	1.89	0.059
		Distance (100 m)	-1.99 ± 0.56	3.53	**0.00042**
		Land use (300 m)	4.98 ± 0.68	7.30	**< 0.0001**
		Row orientation (perpendicular)	0.89 ± 0.33	2.70	**0.0069**
Honey bees	BC	(Intercept)	0.70 ± 0.27	2.57	**0.01**
		Land use (300 m)	12.22 ± 2.52	4.82	**< 0.0001**
	MI	(Intercept)	2.48 ± 1.04	2.39	**0.017**

In Michigan, honey bee abundance on blueberry flowers was negatively correlated with increasing semi-natural habitat at the intermediate scale (1000 m radius; Tables 1 and 2; Fig 7) in the model with the lowest AIC_c_ score, but it was not significantly better than the null model (ΔAIC_c_ = 1.49) or a model using the local habitat scale (300 m) (Table 1). Surprisingly, stocking rate was not predictive of the abundance of honey bees, appearing as an explanatory variable only in suboptimal models (ΔAIC_c_ > 2) (Table 1), and was not a significant variable in model averages (Table 2). However, stocking rates did not vary greatly (4.9–9.4 hives/ha) among Michigan blueberry fields. In contrast to Michigan, honey bee abundance on crop flowers in British Columbia blueberry fields was positively correlated with increasing semi-natural habitat at the local scale (300 m), but counterintuitively this was also not strongly associated with stocking rate, which varied greatly among fields (0–39.5 hives/ha).

### Blueberry pollination and pollen limitation

**Fig 8:**
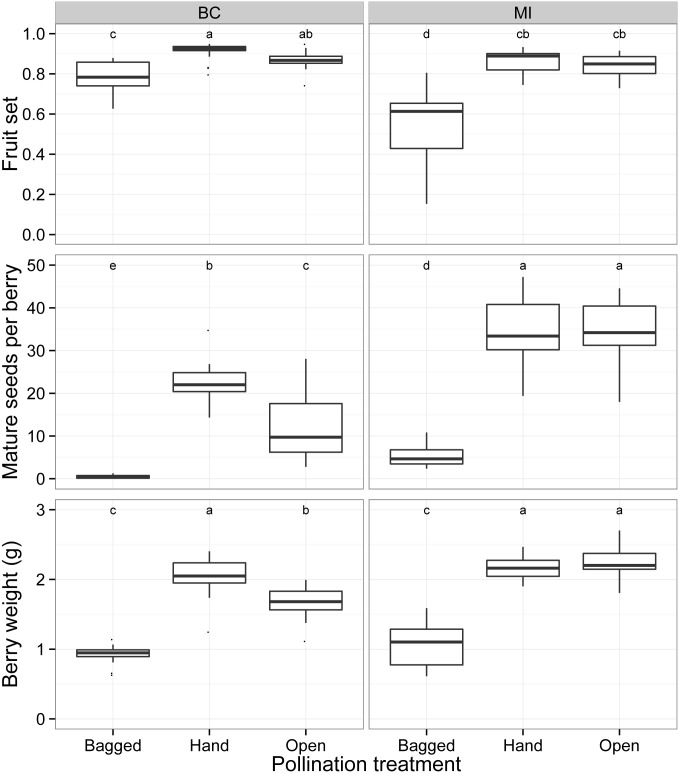
Fruit set, seed set and berry weight across pollination treatments. Comparison of average proportion of set fruit, average number of mature seeds set and average berry weights in sampled clusters for pollinator-excluded (bagged), hand-supplemented pollination (hand) and open to pollinator (open) treatments in British Columbia (BC) and Michigan (MI). Tukey HSD indicated with letters. Hand-supplemented pollination treatments show significant effects on fruit weight (M-W, *U* = 37, *p* < 0.003) and seed set (M-W, *U* = 38, *p* < 0.004) in British Columbia.

**Fig 9:**
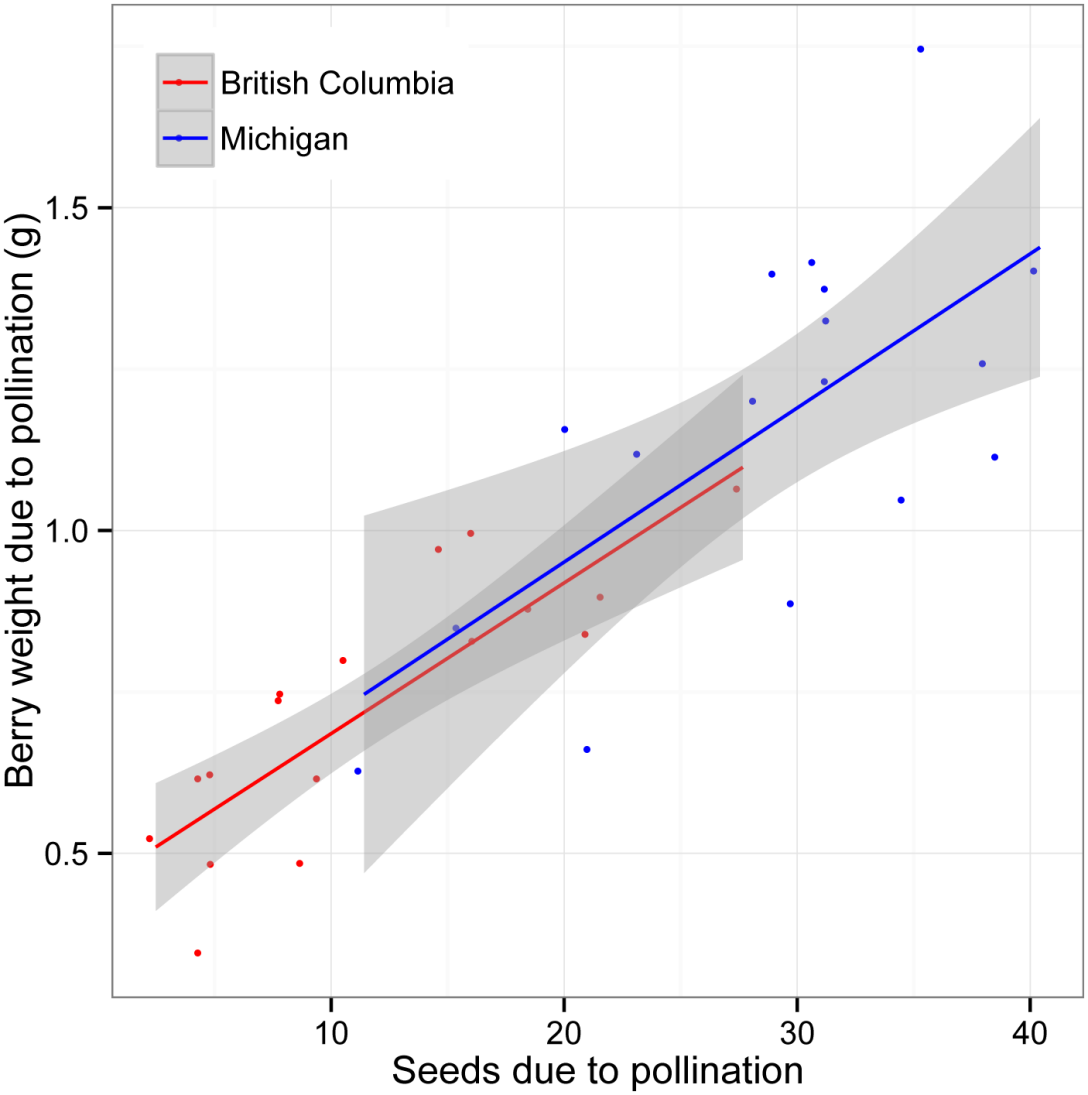
Pollination effects on berry weights. Relationship of the average number of set seeds per berry and the average berry weight in grams, both based on difference between open and pollinator-excluded treatments. The differences between the two pollination treatments were used to determine the number of set seeds and resultant berry weight that were due to pollination by flower-visitors.

In both regions, there was a significant increase in percent fruit set (M-W- Tukey HSD, BC: *U* = 54, *p* < 0.03; MI: *U* = 10, *p* < 0.001), seed set (M-W- Tukey HSD, BC: *U* = 0, *p* < 0.001; MI: *U* = 0, *p* < 0.001) and fruit weight (M-W- Tukey HSD, BC: *U* = 1, *p* < 0.001; MI: *U* = 0, *p* < 0.001) in the open pollination treatment compared with clusters that were bagged throughout bloom (Fig 8), although in BC the effect of pollinator exclusion on fruit set was significantly less than in MI (M-W, *U* = 29, *p* < 0.001). We found different levels of pollen limitation between the two regions, with a significant increase in average berry weight (22% increase, M-W- Tukey HSD: *U* = 37, *p* < 0.003) and seed set (79% increase, M-W- Tukey HSD: *U* = 38, *p* < 0.004) when flowers received hand-supplementation in British Columbia but not in Michigan (Fig 8; M-W; berry weight: *U* = 175, *p* > 0.3; seed set: *U* = 147, *p* > 0.9). In British Columbia, we performed two more hand pollination applications than in Michigan, but since both open and hand-supplemented pollination treatments were exposed to the ambient pollination environment, and seed set and fruit weight in the BC hand-supplemented pollination treatment did not surpass the open treatment in Michigan, the number of hand-applications of pollen are unlikely to be the cause of regional differences (Fig 8). Using the difference between seed set and berry weight between the open and bagged treatments, our results show that the relationship of pollination, measured by seed set, to berry weight is consistent across regions (Fig 9). Analysis of covariance indicates no significant interaction between region and the effect of seeds on berry weight (*F* = 0.056, *p* > 0.8) and the intercepts between the two regions are consistent (BC: 0.48, SE = 0.13; MI: 0.46, SE = 0.18).

We examined the response of blueberry pollination in both regions to the abundance of honey bees and wild bees. Due to autocorrelation between explanatory variables we included all wild bees together and species richness was examined separately from abundance measures. In Michigan, model selection based on AIC_c_ revealed that honey bee abundance on bushes was the primary factor affecting fruit set, fruit weight and seed set (Table 3). In contrast, wild bee abundance was the primary factor affecting fruit weight and seed set in British Columbia, and a model including only bumble bees had lower AIC_c_ scores than when all wild bees were included, indicating that bumble bee abundance explained the data better than did the overall wild bee abundance. Fruit set models for British Columbia did not show any significant differences between the null model and those including honey bees, wild bees, or bumble bees, suggesting that proportion of fruit set is not predicted by bee abundance in these fields. This is consistent with previous studies that have shown some level of parthenocarpy in highbush blueberry [51], but regional differences in fruit set may be due to unmeasured horticultural and nutritional factors.

**Table 3:** Generalized linear models (Gaussian error distribution) examining effects of bee diversity on fruit set, berry weight and seed set benefits due to pollination. Full models included honey bee and wild bee abundance and Chao1 estimated bee richness for Michigan (MI) and Shannon diversity (H) for British Columbia (BC), with interaction terms for each. Among correlated explanatory variables the only the one which showed the best AICc and pseudo-R2 were used. Models with ΔAIC_c_ ≤ 3 are not shown.

Response variable	Region	Explanatory variables	df	logLik	AIC_c_	ΔAIC_c_	Model weight
fruit set gain	MI*	Honey bees	3	14.481	-20.96	0	0.57
		Honey bees + Chao1 richness	4	15.36	-19.07	1.89	0.22
	BC	[null]	2	18.75	-32.16	0	0.47
		H	3	19.81	-30.62	1.54	0.22
		Wild bees	3	19.21	-29.42	2.74	0.12
fruit weight benefit	MI	Honey bees	3	2.62	2.76	0	0.57
		Honey bees x wild bees	5	5.32	5.35	2.59	0.16
	BC	Wild bees	3	4.99	-0.97	0	0.33
		Wild bees + H	4	6.97	-0.22	0.75	0.22
		H	3	4.30	0.40	1.37	0.16
		(null)	2	2.32	0.69	1.66	0.14
seed set	MI	Honey bees	3	-49.19	106.39	0	0.58
		Honey bees + wild bees	4	-48.34	108.32	1.93	0.23
	BC	Wild bees*	3	-32.25	73.50	0	0.75

Asterisks indicate deviation from normality in the response variable (Shapiro-Wilk: W = 0.88, *p* = 0.038) or residuals (Shapiro-Wilk: W = 89, *p* = 0.048).

### Economic benefits of pollination

Yields from open pollinated flowers were estimated to be 14,057 ± 1,112 kg/ha in Michigan and 12,203 ± 1,161 kg/ha in British Columbia (Table 4). If pollinators were excluded, we estimate that yields would decrease by 9,345 ± 1,115 kg/ha in MI and 6,259 ± 812 kg/ha in BC. When examining the clusters that were open to pollinators, fields in Michigan obtained on average a higher proportion of the estimated maximum yield (0.94) than did British Columbia (0.74). Based on mean farm gate prices for 2013, it is estimated that if full pollination could be achieved, i.e. that measured from the pollen-supplemented clusters, on average Michigan growers could increase the total value of their crop by only 6 ± 2 percent, whereas British Columbia growers could obtain significantly greater increases of 38 ± 6 percent over their current crop value (Table 4).

**Table 4:** Economic value of pollinator contributions to yield in British Columbia and Michigan blueberry fields, and potential for economic gains.

Region	BC	MI
Yield estimate per field (kg/ha)	12203 ± 1161	14057 ± 1112
Yield due to pollination per field (kg/ha)	6259 ± 812	9345 ± 1115
Unrealized yield based on hand-supplementation experiment (kg/ha)	4538 ± 692	967 ± 358
Proportion of estimated max yield currently measured	0.74 ± 0.03	0.94 ± 0.02
Mean farm gate price for 2013 ($/kg)	3.30	2.84
Estimated crop value ($/ha)	40270 ± 3832	39920 ± 3157
Estimated value of pollination per field ($/ha)	20655 ± 2679	26541 ± 3167
Potential increase in crop value with maximum pollination ($/ha)	14977 ± 2286	2745 ± 1016
Proportion of potential increase in total value	0.38 ± 0.06	0.06 ± 0.02

## Discussion

There has been significant expansion of highbush blueberry from its native range in eastern North America [52] and it is now being cultivated globally. As such, the community of wild bees visiting this crop can vary considerably across production regions. In the native range of highbush blueberry there is a diversity of wild bee species that visit the flowers, including multiple *Vaccinium-*specialists (e.g. *Andrena carolina* Viereck, *Andrena bradleyi* Viereck, *Colletes validus* Cresson and *Osmia virga* Sandhouse) [53–55]. Although there are wild *Vaccinium* species in British Columbia [56], there are relatively few *Vaccinium*-specialist bees in the region (but see [57]). In the natural range of wild highbush blueberry, and where there has been commercial production for over seventy years, we found a bee fauna with greater species richness and diversity than in British Columbia where this is a relatively recently-introduced crop species. The most abundant wild bee species found in Michigan was the *Vaccinium-*specialist *A*. *carolina*, which agrees with earlier surveys in the state [54]. For bees such as *A*. *carolina* that specialize on a specific plant genus, greater crop production in the landscape may increase their abundance [55], even though loss of natural habitat overall can have a negative effect on bee community abundance and richness [58]. The combination of greater semi-natural habitat and a wild bee community adapted to *V*. *corymbosum* may partially explain the greater species richness and diversity observed in Michigan.

The abundance of wild bees observed per field was similar across the two regions, but the wild bee community observed in British Columbia fields is almost entirely comprised of four species of bumble bee that are native to western North America. Bumble bees are efficient pollinators of blueberry, so pollination services by wild bees in British Columbia may actually be greater despite a less diverse bee community than in Michigan. This would be consistent with recent studies suggesting that bee diversity *per se* is not driving pollination service in crop systems [59,60]. Bumble bee abundance is the most important factor in blueberry pollination in British Columbia based on previous research [16,61] and this is further supported by our study, indicating that bumble bee conservation should be a priority for growers in British Columbia wishing to increase and diversify their pollination services.

In addition to finding a varying community of wild bees, honey bees were found to be far more abundant in Michigan than in British Columbia, and despite the diversity of wild bees present honey bees were the best predictor of pollination levels in Michigan. Honey bee abundance on flowers was not strongly related to stocking density (0–39.5 hives/ha), although stocking rate was included in some of the suboptimal models (Table 1). This was true even for British Columbia, where stocking rates were higher than in Michigan, and can range up to 8 times the provincially recommended amount [14]. Although a lack of relationship between stocking density and honey bee abundance seems counterintuitive, similar results have been reported in other areas of blueberry production [7]. Blueberry is considered a relatively poor resource for honey bees, and colonies can decline when foraging on blueberry [62]. The poor correlation between stocking rate and abundance within fields, and the association between honey bee abundance and various landscape scales (see below), suggests that honey bees are foraging on other resources outside of the focal fields [63]. This could include adjacent blueberry fields with more accessible and rewarding cultivars [64], other more attractive crops, or flowers in natural habitat. Additionally, colonies in nearby fields were not surveyed, and honey bees from neighboring colonies could be visiting if the field is rewarding and within their flight range. Taken together, our results suggest colony number may not be a good predictor of the foraging forces of honey bees in a field and so increasing honey bee stocking rates is not necessarily sufficient to resolve pollination deficits in highbush blueberry. Instead we suggest that monitoring colony strength and abundance at a larger spatial scale may be necessary to understand patterns of honey bee pollination.

In Michigan, honey bee abundance on bushes was negatively correlated with increasing proportion of semi-natural habitat in the surrounding landscape, although the correlation is weak based on AICc values. The observed trend supports the idea that honey bees are foraging on alternative resources available in the area [62,63]. The relative value of blueberry bloom to long-distance foragers such as honey bees would decrease with increasing availability of alternative resources in the surrounding landscape. In British Columbia, honey bee abundance was also negatively correlated with semi-natural habitat in the landscape (2000 m radius; Fig 7), but counterintuitively there was a stronger positive correlation with increasing semi-natural habitat in the local area (300 m radius). This apparent contradiction could be explained if the alternative resources in the area adjacent to British Columbia blueberry fields draw honey bees into the general vicinity of the fields. Honey bee visits to blueberry are primarily for nectar [63], but visits may be more frequent to fields if there are also nearby pollen resources. The extent of semi-natural habitat surrounding blueberry fields at the landscape scale is typically much lower in British Columbia than in Michigan (Figs 1, 2 and 7) so local pollen resources may of greater relative importance for honey bee colonies in British Columbia.

The varying response of the wild bee communities to local and landscape resources in the two regions reflects the community composition and their different foraging abilities and preferences [17,18]. In Michigan, the bee community comprises mainly smaller, solitary species (S3 Table) that respond positively to local scale resources. These bees would be exploiting nesting and foraging resources in field margins and nearby wooded habitat, also explaining the decline in abundance with distance from the field edge. The positive response of wild bees to rows perpendicular to the field edge suggest that bees are more likely to move along rows of blueberry bushes rather than cross them, a behavioral trait that has been highlighted for honey bees [65]. In contrast, the community dominated by bumble bees in British Columbia was positively correlated with natural habitat at the larger landscape scale (2000 m radius; Fig 7), reflecting their much larger foraging range [18]. The weaker effect of local scale factors on wild bees in British Columbia indicates that bees visiting these fields are limited more by nesting and floral resources at the larger scales and supports recent analyses showing that life history traits affect how bees respond to local and landscape factors [66]. The much lower levels of semi-natural habitat present at the local scale in British Columbia may also make resources at other spatial scales more important for bumble bees which can exploit them via their stronger flight ability. This highlights the importance of considering the needs and foraging ranges of the fauna to be conserved in agroecosystems, as well as land use strategies at both local and landscape scales [5].

The contrasting communities of bees and their responses to habitat quality at different scales also suggests that efforts to add habitat for supporting bees within farms may need different strategies across blueberry-growing regions. Small, univoltine, wild bees visiting blueberry in Michigan include *Vaccinium*-specialists and floral generalists that may be limited by floral resources just before or after blueberry bloom, suggesting a conservation strategy focused on providing floral resources at these specific times may be effective in these locations. In contrast, enhancement of bumble bees that are either at relatively low population levels (Michigan) or responsive only to landscape-scale habitat (British Columbia) may require different approaches that provide floral resources throughout the season after blueberry bloom is complete [67]. Providing support to this idea, establishing habitat adjacent to Michigan blueberry fields has recently been shown to enhance abundance of social and solitary groups of bees, while also enhancing blueberry pollination and other beneficial insects and their ecosystem services [68,69].

The importance of bumble bees for blueberry pollination in British Columbia, and the superior individual effectiveness of bumble bee queens as pollinators of blueberry in comparison to honey bees [7,12,70], suggests that managed bumble bee colonies may be an effective strategy to improve pollination of highbush blueberries where pollination deficits occur. Managed colonies of *Bombus impatiens* Cresson are already available in eastern North America for blueberry pollination [50,71–73] and there are ongoing efforts to commercialize western bumble bees for pollination [74]. Managed bumble bees have been shown to be effective alternative pollinators of some specialty crops [75,76], and studies are needed to determine the effectiveness and economic suitability of managed bumble bee colonies for supplementing honey bees in highbush blueberry. There is some concern that managed bumble bee colonies could be detrimental to wild populations through pathogen spillover [67,77,78] and additional study is needed to determine the extent of this threat to wild bee populations.

The economic consequences of sub-optimal pollination can be substantial. Large pollination deficits were found in British Columbia blueberry fields, and deficits decreased (and profits increased) largely due to the activity of wild bumble bees. In contrast, Michigan fields had minimal pollination deficits and profit accrues primarily due to honey bee pollination of the crop, in spite of the high diversity of wild bees on farms. The value of the economic benefit depends on the price per kilogram and the assumption that individual berry weights correlate with field-level yields. Our sites were intensively managed commercial fields with yields which often surpass local averages [13,79], so the effect of pollen-limitation on yield in less intensively managed farms may differ. Our results, which suggest that pollination-dependent yield is most impacted either by abundance of honey bees in Michigan or a few species of bumble bees in British Columbia, are consistent with other studies which suggest a small subset of the pollinator community is responsible for delivering the majority of pollination ecosystem services [59,60].

To fully understand the extent of pollen limitation in highbush blueberry, additional studies are needed which allow comparison across multiple growing regions. However, a number of management strategies show promise for increasing economic gains from pollination. Honey bees remain the primary, and are currently the most economical, method of increasing local bee abundance with minimal effort. As our results demonstrate, there may be diminishing returns for blueberry yield with increased investment in honey bees, as stocking rate is not strongly associated with pollination levels. There are several cases where other managed bees are more effective crop pollinators than honey bees [20], including *Megachile rotundata* (Fabricius) in alfalfa [80,81], bumble bees in tomato [73], and mason bees (*Osmia* spp.) in pome fruits [82,83]. The economic viability of using additional managed bees to support crop pollination, including bumble bees in blueberry fields, needs further study. Wild bees also provide significant pollination services [84] and habitat management strategies can be used to help conserve and increase their abundance [68]. Wildflower plantings which provide alternative forage outside of crop bloom can be of particular benefit for both multivoltine solitary bee species and eusocial bumble bees and sweat bees. Habitat enhancements have high initial costs but have the potential to increase unmanaged pollination services to a level that results in a measurable return on investment [68]. Furthermore, our results suggest that row orientation plays a role in the movement of bees in crop fields. This result has implications for how new blueberry plantings are arranged in relation to existing semi-natural habitat and also the optimal placement of habitat enhancements relative to existing blueberry fields. Additional studies to examine the effectiveness of combinations of these diverse strategies are needed to better document the long term stability of crop pollination across a wider geographic context [85].

## Supporting Information

S1 Table Blueberry field study sites in British Columbia and Michigan. List of blueberry fields used as study sites with grower supplied values for honey bee stocking rates and hive rental cost estimates. Hive rental costs from Canada have been converted to USD based on mean 2013 exchange rate of CAD (0.97). To protect identity of growers latitude and longitude are only approximate values.
(DOCX)

S2 Table Land use categories. Pre-determined land use categories used in hand-digitization of 300 m radius surrounding focal blueberry fields. Not all categories were observed in actual radii.
(DOCX)

S3 Table Blueberry flower-visitors in British Columbia and Michigan. List of insects recorded visiting blueberry flowers in commercial highbush blueberry (*Vaccinium corymbosum* L., cv. Bluecrop) fields in British Columbia (BC) and Michigan (MI) during timed (10 min.) samples in 2013.
(DOCX)
