# Supplementary material for: Contrasting Pollinators and Pollination in Native and Non-Native Regions of Highbush Blueberry Production
Source: PLoS One. 2016 Jul 8;11(7):e0158937. doi: 10.1371/journal.pone.0158937 (PMC4938509; doi:10.1371/journal.pone.0158937)
Supplement: S1 Table — List of blueberry fields used as study sites with grower supplied values for honey bee stocking rates and hive rental cost estimates. Hive rental costs from Canada have been converted to USD based on mean 2013 exchange rate of CAD (0.97). To protect identity of growers latitude and longitude are only approximate values. (DOCX) [file pone.0158937.s001.docx]

**S1 Table.** **Blueberry field study sites in British Columbia and Michigan.** List of blueberry fields used as study sites with grower supplied values for honey bee stocking rates and hive rental cost estimates. Hive rental costs from Canada have been converted to USD based on mean 2013 exchange rate of CAD (0.97). To protect identity of growers latitude and longitude are only approximate values.

| State/Province | Blueberry Field | Latitude | Longitude | Stocking rate  (hives per ha) | Price per hive (USD) |
| --- | --- | --- | --- | --- | --- |
| BC | BCsite1 | 49.1 | -122.3 | 9.9 | NA |
| BC | BCsite2 | 49.1 | -122.3 | 14.8 | 102 |
| BC | BCsite3 | 49.1 | -122.3 | 9.9 | ~97 |
| BC | BCsite4 | 49.0 | -122.4 | 0 | NA |
| BC | BCsite5 | 49.1 | -122.4 | 9.9 | NA |
| BC | BCsite6 | 49.1 | -122.4 | 9.9 | NA |
| BC | BCsite7 | 49.1 | -122.9 | 14.8 | 107 |
| BC | BCsite8 | 49.1 | -122.9 | 14.8 | 107 |
| BC | BCsite9 | 49.3 | -122.6 | 9.9 | 97–126 |
| BC | BCsite10 | 49.2 | -122.7 | 9.9 | 107 |
| BC | BCsite11 | 49.3 | -122.6 | 39.5 | 116 |
| BC | BCsite12 | 49.3 | -122.7 | 39.5 | 116 |
| BC | BCsite13 | 49.3 | -122.7 | 9.9 | 116 |
| BC | BCsite14 | 49.2 | -122.7 | 9.9 | 87–116 |
| BC | BCsite15 | 49.1 | -122.8 | 9.9 | NA |
| BC | BCsite16 | 49.1 | -122.8 | 9.9 | 116 |
| BC | BCsite17 | 49.1 | -122.8 | 7.4 | 58–97 |
| MI | MIsite1 | 43 | -86.1 | 7.4 | 65 |
| MI | MIsite2 | 42.5 | -86 | 6.4 | 50 |
| MI | MIsite3 | 42.3 | -86.1 | 8.2 | 50 |
| MI | MIsite4 | 42.3 | -85.8 | 7.4 | 55 |
| MI | MIsite5 | 42.9 | -86.2 | 6.4 | 53 |
| MI | MIsite6 | 42.4 | -85.9 | 6.8 | 54 |
| MI | MIsite7 | 42.8 | -86.2 | 7.4 | 65 |
| MI | MIsite8 | 42.3 | -85.9 | 7.4 | 55 |
| MI | MIsite9 | 42.3 | -85.8 | 7.4 | 55 |
| MI | MIsite10 | 42.4 | -86.1 | 6.9 | 50 |
| MI | MIsite11 | 42.4 | -86.1 | 9.4 | 50 |
| MI | MIsite12 | 42.9 | -86.2 | 7.4 | 65 |
| MI | MIsite13 | 42.3 | -85.9 | 6.8 | 55 |
| MI | MIsite14 | 42.9 | -86.1 | 4.9 | 54 |
| MI | MIsite15 | 42.4 | -86.1 | 7.4 | 75 |
| MI | MIsite16 | 42.4 | -86.1 | 6.4 | 50 |
| MI | MIsite17 | 42.4 | -85.9 | 6.8 | 54 |
