# Supplementary material for: Contrasting Pollinators and Pollination in Native and Non-Native Regions of Highbush Blueberry Production
Source: PLoS One. 2016 Jul 8;11(7):e0158937. doi: 10.1371/journal.pone.0158937 (PMC4938509; doi:10.1371/journal.pone.0158937)
Supplement: S2 Table — Pre-determined land use categories used in hand-digitization of 300 m radius surrounding focal blueberry fields. Not all categories were observed in actual radii. (DOCX) [file pone.0158937.s002.docx]

**S2 Table.** **Land use categories.** Pre-determined land use categories used in hand-digitization of 300 m radius surrounding focal blueberry fields. Not all categories were observed in actual radii.

| Land use categories | Land use explanation | Land use group |
| --- | --- | --- |
| Annual crop, non-flowering | Cereal and grain crops | Disturbed/Agriculture |
| Annual crop, flowering | Flowering crops, e.g. sunflowers | Disturbed/Agriculture |
| Perennial crop, non-flowering | Orchards, non-rewarding for bees | Disturbed/Agriculture |
| Perennial crop, flowering | Orchards, rewarding for bees, e.g. apple or cherry | Disturbed/Agriculture |
| Bare | Non-vegetated ground cover | Disturbed/Agriculture |
| Marshland | Marshes and vegetated wetlands | Semi-natural |
| Mown grass | Lawns, mown roadsides | Disturbed/Agriculture |
| Grazed pasture | Pasture for livestock | Disturbed/Agriculture |
| Grazed savanna | Savanna grazed by livestock | Disturbed/Agriculture |
| Savanna, ungrazed | Savanna, undisturbed understory | Semi-natural |
| Sparse, weedy scrub | Open areas with undisturbed weeds | Semi-natural |
| Shrub | Shrubland | Semi-natural |
| Woodland, open | open woodland | Semi-natural |
| Woodland, deciduous | Woods dominated by deciduous trees | Semi-natural |
| Woodland, coniferous | Woods dominated by coniferous trees | Semi-natural |
| Woodland, mixed | Woods with both deciduous and coniferous trees in abundance | Semi-natural |
| Pavement | roads: paved or heavily driven dirt | Disturbed/Agriculture |
| High intensity, development | Industrial areas, urban zones | Disturbed/Agriculture |
| Low intensity, open development | Suburban areas, high density | Disturbed/Agriculture |
| Low intensity, wooded development | Human dwellings in wooded areas | Disturbed/Agriculture |
| Low density, open development | Suburban areas, low density | Disturbed/Agriculture |
| Water | Natural and manmade bodies of water | Disturbed/Agriculture |
| Riparian, open | Riparian zones with short vegetation | Semi-natural |
| Riparian, wooded | Riparian zones in wooded areas | Semi-natural |
| Planted, non-agriculture | Managed plants, e.g., hedges | Disturbed/Agriculture |
| Planted, native | Managed native plant restorations | Semi-natural |
