# Supplementary material for: Contrasting Pollinators and Pollination in Native and Non-Native Regions of Highbush Blueberry Production
Source: PLoS One. 2016 Jul 8;11(7):e0158937. doi: 10.1371/journal.pone.0158937 (PMC4938509; doi:10.1371/journal.pone.0158937)
Supplement: S3 Table — List of insects recorded visiting blueberry flowers in commercial highbush blueberry (Vaccinium corymbosum L., cv. Bluecrop) fields in British Columbia (BC) and Michigan (MI) during timed (10 min.) samples in 2013. (DOCX) [file pone.0158937.s003.docx]

**S3 Table.** **Blueberry flower visitors in British Columbia and Michigan.** List of insects recorded visiting blueberry flowers in commercial highbush blueberry (*Vaccinium corymbosum* L., cv. Bluecrop) fields in British Columbia (BC) and Michigan (MI) during timed (10 min.) samples in 2013.

| Order | Family | Species | Author | Region |
| --- | --- | --- | --- | --- |
| Hymenoptera | Andrenidae | *Andrena bradleyi* | Viereck, 1907 | MI |
| Hymenoptera | Andrenidae | *Andrena carlini* | Cockerell, 1901 | MI |
| Hymenoptera | Andrenidae | *Andrena carolina* | Viereck, 1909 | MI |
| Hymenoptera | Andrenidae | *Andrena cressonii* | Robertson, 1891 | MI |
| Hymenoptera | Andrenidae | *Andrena hemileuca* | Viereck, 1904 | BC |
| Hymenoptera | Andrenidae | *Andrena imitatrix* | Cresson, 1872 | MI |
| Hymenoptera | Andrenidae | *Andrena miserabilis* | Cresson, 1872 | MI |
| Hymenoptera | Andrenidae | *Andrena nivalis* | Smith, 1853 | BC |
| Hymenoptera | Andrenidae | *Andrena pruni* | Robertson, 1891 | MI |
| Hymenoptera | Andrenidae | *Andrena rugosa* | Robertson, 1891 | MI |
| Hymenoptera | Andrenidae | *Andrena heraclei** | Robertson, 1897 | MI |
| Hymenoptera | Andrenidae | *Andrena vicina* | Smith, 1853 | MI |
| Hymenoptera | Apidae | *Bombus bimaculatus* | Cresson, 1863 | MI |
| Hymenoptera | Apidae | *Bombus flavifrons* | Cresson, 1863 | BC |
| Hymenoptera | Apidae | *Bombus griseocollis* | (DeGeer, 1773) | MI |
| Hymenoptera | Apidae | *Bombus impatiens* | Cresson, 1863 | BC, MI |
| Hymenoptera | Apidae | *Bombus melanopygus* | Nylander, 1848 | BC |
| Hymenoptera | Apidae | *Bombus mixtus* | Cresson, 1878 | BC |
| Hymenoptera | Apidae | *Bombus vagans* | Smith, 1854 | MI |
| Hymenoptera | Apidae | *Bombus vosnesenskii* | Radoszkowki, 1862 | BC |
| Hymenoptera | Apidae | *Ceratina calcarata* | Robertson, 1900 | MI |
| Hymenoptera | Apidae | *Ceratina mikmaqi* | Rehan and Sheffield, 2011 | MI |
| Hymenoptera | Apidae | *Nomada* cf. *lepida* | Cresson, 1863 | MI |
| Hymenoptera | Apidae | *Nomada luteoloides* | Robertson, 1895 | MI |
| Hymenoptera | Apidae | *Nomada* sp. |  | MI |
| Hymenoptera | Apidae | *Xylocopa virginica* | (Linnaeus, 1771) | MI |
| Hymenoptera | Halictidae | *Augochlora pura* | (Say, 1837) | MI |
| Hymenoptera | Halictidae | *Halictus ligatus* | Say, 1837 | MI |
| Hymenoptera | Halictidae | *Lasioglossum acuminatum* | McGinley, 1986 | MI |
| Hymenoptera | Halictidae | *Lasioglossum coriaceum* | (Smith, 1853) | MI |
| Hymenoptera | Halictidae | *Lasioglossum hitchensi* | Gibbs, 2012 | MI |
| Hymenoptera | Halictidae | *Lasioglossum leucocomum* | (Lovell, 1908) | MI |
| Hymenoptera | Halictidae | *Lasioglossum leucozonium* | (Schrank, 1781) | MI |
| Hymenoptera | Halictidae | *Lasioglossum obscurum* | (Robertson, 1892) | MI |
| Hymenoptera | Halictidae | *Lasioglossum subviridatum* | (Cockerell, 1938) | MI |
| Hymenoptera | Halictidae | *Lasioglossum versans* | (Lovell, 1905) | MI |
| Hymenoptera | Halictidae | *Lasioglossum zonulum* | (Smith, 1848) | BC |
| Hymenoptera | Megachilidae | *Osmia atriventris* | Cresson, 1864 | MI |
| Hymenoptera | Megachilidae | *Osmia bucephala* | Cresson, 1864 | MI |
| Hymenoptera | Megachilidae | *Osmia pumila* | Cresson, 1864 | MI |
| Hymenoptera | Tiphiidae | *Tiphia* sp*.* |  | MI |
| Hymenoptera | Vespidae | *Vespula acadica* | (Sladen, 1918) | BC |
| Diptera | Calliphoridae | *Lucilia illustris* | (Meigen, 1826) | BC |
| Diptera | Calliphoridae | *Lucilia* sp. |  | MI |
| Diptera | Syrphidae | *Allograpta obliqua* | (Say, 1823) | MI |
| Diptera | Syrphidae | *Eristalis dimidiata* | (Wiedemann, 1830) | MI |
| Diptera | Syrphidae | *Eristalis flavipes* | Walker, 1849 | BC |
| Diptera | Syrphidae | *Eristalis stipator* | Osten Sacken, 1877 | MI |
| Diptera | Syrphidae | *Eristalis tenax* | (Linnaeus, 1758) | BC |
| Diptera | Syrphidae | *Eupeodes lapponicus* | (Zetterstedt, 1838) | BC |
| Diptera | Syrphidae | *Eupeodes latifasciatus* | (Macquart, 1829) | BC, MI |
| Diptera | Syrphidae | *Helophilus latifrons* | Loew, 1863 | BC, MI |
| Diptera | Syrphidae | *Lejops s*p. |  | BC |
| Diptera | Syrphidae | *Platycheirus* sp. |  | BC |
| Diptera | Syrphidae | *Sphaerophoria pyrrhina* | Bigot, 1884 | MI |
| Diptera | Syrphidae | *Syritta pipiens* | (Linnaeus, 1758) | BC |
| Diptera | Syrphidae | *Syrphus opinator* | Osten Sacken, 1877 | BC |
| Diptera | Syrphidae | *Toxomerus geminatus* | (Say, 1823) | MI |
| Diptera | Syrphidae | *Toxomerus marginatus* | (Say, 1823) | MI |
| Diptera | Tabanidae | *Hybomitra lasiophthalma* | (Macquart, 1838) | MI |
| Diptera | Tachinidae | *Epalpus signifer* | (Walker, 1849) | MI |

* New record for Michigan State
